# Editorial: Sensorimotor decoding: characterization and modeling for rehabilitation and assistive technologies

**DOI:** 10.3389/fnhum.2023.1243226

**Published:** 2023-07-18

**Authors:** Miguel Pais-Vieira, Tetiana Aksenova, Vassiliy Tsytsarev, Jean Faber

**Affiliations:** ^1^Department of Medical Sciences, iBiMED—Institute of Biomedicine, University of Aveiro, Aveiro, Portugal; ^2^Univ. Grenoble Alpes, CEA, LETI, Clinatec, Grenoble, France; ^3^School of Medicine, University of Maryland, Baltimore, MD, United States; ^4^Division of Neuroscience, Department of Neurology and Neurosurgery, Escola Paulista de Medicina, Federal University of São Paulo, São Paulo, Brazil

**Keywords:** sensorimotor decoding, brain-computer interface (BCI), brain-machine interface (BMI), rehabilitation, assistive technology, neurophysiological mechanisms, classification models

Understanding how sensory information is encoded, processed and related to motor actions is fundamental for the development of effective rehabilitation protocols and support technologies, such as neural modulation procedures and brain-machine interfaces. Currently there are different mathematical and computational methods that seek to elucidate these questions. Each strategy presents a particular way of approaching the problem of sensorimotor coding and a different solution of application (Figure 1).

**Figure 1:**
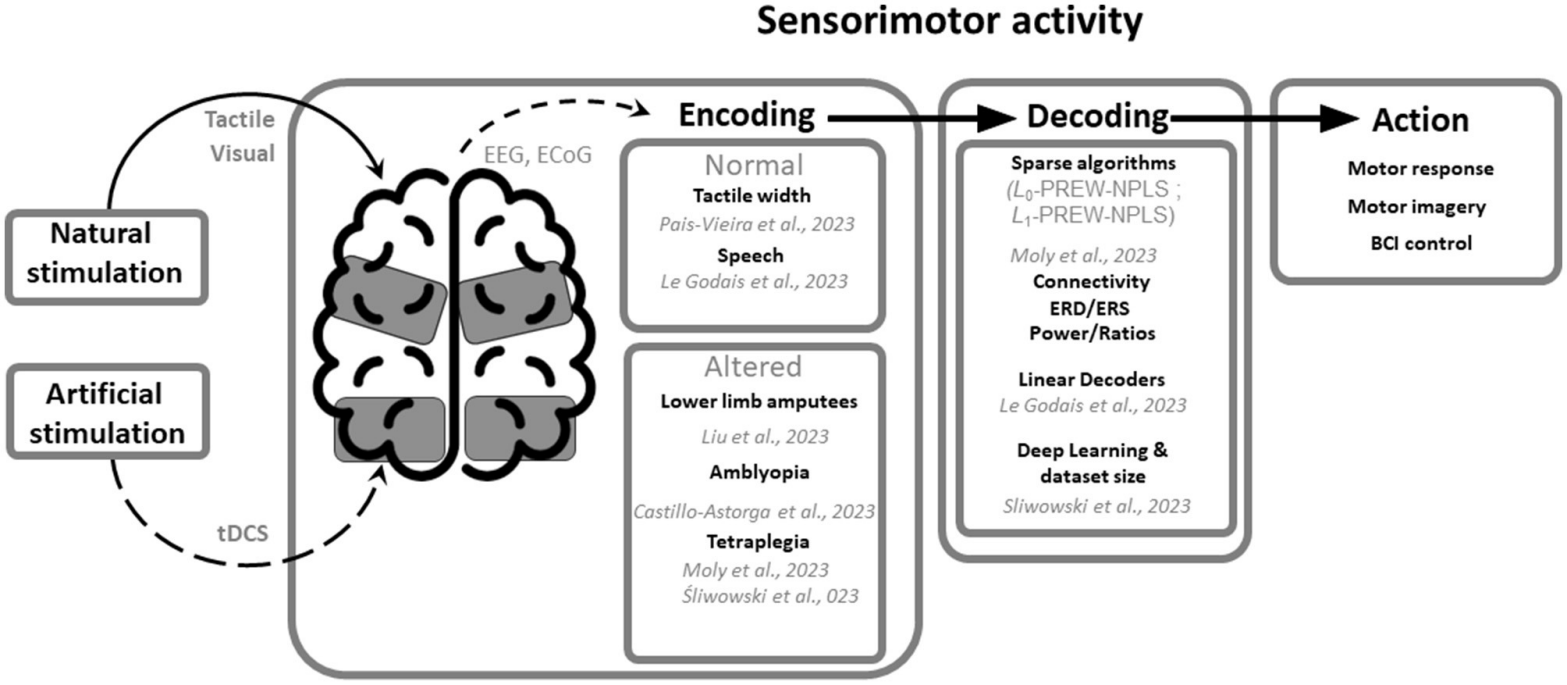
Advancements in the characterization and modeling of neural activity in the Research Topic sensorimotor decoding. The Research Topic on sensorimotor activity has brought together studies that utilize natural and artificial stimulation, providing new insights into the encoding of sensorimotor activity under normal and altered conditions. Furthermore, various decoding approaches, including sparse algorithms, connectivity analysis, ERD/ERS analysis, power and power ratios, linear decoders and deep learning, have been employed to decode sensorimotor activity and, ultimately, generate motor responses, motor imagery, and BCI control.

The present Research Topic on Sensorimotor Decoding aimed to increase our current understanding of sensorimotor processing in healthy individuals and in pathologies to translate this knowledge to rehabilitation protocols, brain-computer interfaces (BCIs), and neurophysiological mechanisms through different approaches applied to the characterization, identification, and classification of sensorimotor patterns.

Liu et al. have studied motor imagery (MI) in people with lower limb amputation with chronic pain. During the MI task subjects had to randomly extend their left or right leg mentally, without producing the actual movement. In this way, the authors were able to compare the mental executions in each situation. They report that people with amputation presented an increase in event-related synchronization (ERS) in the mu band while control subjects presented a significant event-related desynchronization (ERD). In addition, phantom limb pain was negatively correlated with the power of mu or low beta frequency bands in electrodes related to sensorimotor activity. This bilaterally MI affects related neural activity in people with amputation raises relevant questions regarding our ability to generalize BCI decoding algorithms (Rodrigues et al., 2022). As some of these changes were correlated to phantom limb pain, this study also constitutes a relevant bridge in our understanding of the neuroplastic changes occurring in people with amputation.

Sensory encoding and neural plasticity were studied in amblyopia by Castillo-Astorga et al.. Although the treatment of amblyopia is well-established for children, in adults neuroplasticity effects are not easy to find. The authors have analyzed the effects of combining transcranial Direct-Current Stimulation (tDCS) and ocular occlusion in adults with amblyopia. Although other studies have used tDCS for amblyopia, the authors have implemented a new approach accounting for: (i) the relevance of the different anatomical stations throughout the visual pathway, (ii) the importance of inter-hemispheric communication, and (iii) effects from the combination of the anatomical constraints of the visual pathway with the predicted effects of anode and cathode electrodes along the different pathways. This study elegantly demonstrates that sensory encoding and neural plasticity can occur as the result of the potentiation between multiple experimental approaches.

Moly et al. have explored the problem of online feature space dimensionality reduction for BCI closed-loop experiments. Embedded feature selection was studied in electrocorticography (ECoG) signals pre-recorded from the sensorimotor cortex of a tetraplegic subject controlling the upper limbs of a virtual avatar in three dimensions. Three proposed algorithms were employed, using L_0_, L_0.5_, L_1_ norm and pseudo-norm penalties, been directly integrated to the tensor factorization procedure of the Recursive Exponentially Waited N-way Partial Least Square (REW-NPLS) algorithm. This allows slice-wise decoder sparsity and significantly reduces dimensionality and computational effort. The study reports equal or better decoding performance when these algorithms were compared to generic REW-NPLS algorithms. Tested in pseudo-online mode the proposed algorithms were demonstrated to be compatible with real-time computations. The authors raise the question of being able to achieve different results if larger data sets had been used (see Śliwowski et al. below). This study also raises the important question of the need to improve decoding associated with frequency and temporal domains.

The study of Śliwowski et al. addressed the impact of dataset size and long-term electrocorticogram (ECoG) based BCI using two deep learning (DL) models (Sliwowski et al., 2022) and a multilinear model. The authors have also addressed the potential for patient adaptation (i.e., neural plasticity). The use of ECoG signals in this study, is of high relevance because they: (i) allow for high temporal resolution signals, (ii) are not affected by skull and scalp attenuation of neural signals, and (iii) are not likely to suffer from displacement (normally happening in repeated EEG recordings). The authors demonstrated that although all three models required the same amount of data, DL models presented higher decoding performance. Another relevant aspect of this study is that the authors have introduced patient-decoder co-adaptation as an integral part of their study design, accounting for the possibility of neural plasticity occurring throughout the implementation of BCIs.

The need for fast decoding algorithms, and specifically for reconstructing speech in real time from ongoing cortical activity, was explored by Le Godais et al.. These authors have compared the performance of several linear decoders for off-line reconstruction of articulatory movements associated with speech reconstruction. These decoders are often used for motor imagery to control upper and lower limbs in BCIs, but not for speech decoding. The authors report similar performances for all three linear decoders (vanilla linear regression, ridge-regularized linear regressions, and partial least squares regressions) therefore supporting the need for additional studies using these decoders in real-time. Although intelligibility was not achieved, it should be highlighted that speech decoding is an especially difficult field to develop BCIs, since it requires millisecond precision accuracy while, for most of BCIs, the time windows span up to 100 ms.

Tactile processing in the field of BCIs and neurorehabilitation has come to be a matter of increasing importance. The study of Pais-Vieira et al. has analyzed EEG activity while subjects discriminated the width between two movable bars. This study is of interest to the present topic because: (i) this is the first detailed description of neural activity in humans performing tactile width discriminations. This is a topic that has been largely described in rodents, but only recently a task was developed to study this type of processing in humans (Perrotta et al., 2020). Second, the authors demonstrate that neural activity in fronto-parietal electrodes encoded between-subjects' performances while parieto-occipital electrodes encoded within-subjects' performances. These results may be useful to test in future BCIs, how changes in power of fronto-parietal-occipital networks affect BCI decoding and performance.

The collection of manuscripts gathered in the present Research Topic suggests that a fine balance between new decoding algorithms and time-proven approaches such as anatomical based therapies, behavioral testing, and neural modulation (Figure 1); can lead to improved rehabilitation strategies and an improved understanding of the basic mechanisms underlying sensorimotor processing.

## Author contributions

MP-V, TA, VT, and JF conceived the Research Topic's idea and wrote the editorial paper. All authors approved and corrected the final version of the manuscript. All authors contributed to the article and approved the submitted version.
